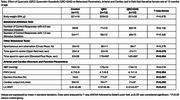# Senolytic treatment with Quercetin and Dasatinib mitigates decline in cardiac function and links heart remodeling to cognitive performance in adult Dahl Salt‐Sensitive Female Rats

**DOI:** 10.1002/alz70855_104317

**Published:** 2025-12-24

**Authors:** Carla Rocha Dos Santos, Mackenzie J Barnett, Ross A McDevitt, Wen Wei, Valentina I Zernetkina, Christopher H Morrell, Edward G Lakatta, Olga V Fedorova

**Affiliations:** ^1^ National Institute on Aging/National Institutes of Health (NIA/NIH), Baltimore, MD, USA

## Abstract

**Background:**

Cardiovascular risk factors, including elevated blood pressure (BP) and central arterial stiffness (CAS), an early vascular aging (EVA) characteristic, are associated with cognitive decline and dementia. In adult Dahl salt‐sensitive (DSS) rats, consuming a normal salt (NS; 0.5% NaCl) diet, EVA and cognitive impairment progress with age. Previously, we demonstrated that senolytic treatment with Quercetin (QRC), a natural flavonoid with antioxidant, anti‐inflammatory, and senolytic properties, combined with Dasatinib (DAS), a tyrosine kinase inhibitor, reduced EVA and improved cognitive function in adult DSS males. We hypothesized that QRC and DAS will affect EVA and heart parameters and improve cognitive function in DSS females.

**Methods:**

Six‐month old DSS female rats were treated with placebo (control group, *n* = 11), or QRC (100 mg/kgBW/day; *n* = 13), or a combination of QRC+DAS (*n* = 12) for 6 months. DAS (1mg/kgBW/day) was added for the final 2 months to the QRC+DAS group. All treatments were supplemented to a standard NS diet. Systolic BP (SBP), pulse wave velocity (PWV, an index of CAS), echocardiography, cognitive function (visuospatial attention test and cross maze), and anxiety‐like behavior (open field and elevated plus maze) were evaluated following treatment or placebo.

**Results:**

At 12 months of age, neither QRC nor QRC+DAS chronic treatments affected SBP or PWV compared to controls. However, both treatments improved heart function, with increased ejection fraction and fractional shortening. QRC+DAS treatment reduced left ventricle relative wall thickness (LV‐RWT) versus controls. Cognitive function did not differ significantly among groups. The rats in both QRC and QRC+DAS groups exhibited reduced anxiety‐like behavior spending more time in open arms. Number of correct attention test responses negatively correlated with LV‐RWT (r=‐0.813, *p* = 0.004) and positively with cardiac output (r=0.760, *p* = 0.02) in the QRC group; and negatively correlated with LV‐RWT (r=‐0.683, *p* = 0.021) and positively correlated with ejection fraction (r=0.577, *p* = 0.063) in the QRC+DAS group, suggesting a link between cardiac structure and attentional performance.

**Conclusions:**

Chronic senolytic treatments with QRC or QRC+DAS protected heart function and reduced anxiety‐like behavior but did not affect EVA in adult DSS females. The correlation between cardiac remodeling and attention test performance highlights a potential heart‐brain interaction.

Supported by NIA/NIH/IRP